# A Communication to the Chief Surgeon of the Department of Santiago and Puerto Principe

**Published:** 1900-03

**Authors:** Bat Smith

**Affiliations:** Yellow Fever Hospital, Santiago Bay


### TEXAS MEDICAL JOURNAL.

AUSTIN, TEXAS.

A MONTHLY JOURNAL OF MEDICINE AND SURGERY.

EDITED AND PUBLISHED BY

F. E. DANIEL, M. D., AND S. E. HUDSON, M. D.

Published Monthly at Austin, Texas, by Drs. Daniel and Hudson. Subscription price $1.00 a year in advance.

Eastern Representative: John Guy Monihan, St. Paul Building, 220 Broadway, New York City.

Official organ of the West Texas Medical Association, the Houston District Medical Association, the Austin District Medical Society, the Brazos Valley Medical Association, the Galveston County Medical Society, and several others.

For Texas Medical Journal.

### A Communication to the Chief Surgeon of the Department of Santiago and Puerto Principe.

YELLOW FEVER HOSPITAL, SANTIAGO BAY,
September 19, 1899.

*Major V. Harvard, U. S. A., Chief Surgeon, Department of Santiago and Puerto Principe.*

SIR: I have the honor to forward this communication for your consideration. Owing to some controversies which have occurred of late, relative to the treatment of yellow fever during this epidemic, I deem it my duty to submit to you, as chief surgeon, my personal views on the subject, as the great majority of the patients treated at this hospital were under my sole care.

A treatment inaugurated by A. A. Surgeon E. F. Nunez, and, which, I said in a former communication to you, deserves due consideration, has found favor with some of the medical officers of the department.

The main feature in this treatment seems to be the doctor's great reliance upon and faith in the therapeutical value of salol, with occasional doses of cream of tartar water accidulated with a little lime juice; also irrigation of the bowels with warm water. Lemonade, hot or cold, is also given according to the necessities of the case.

Dr. Nunez argues that salol acts as an antiseptic, rendering the stools absolutely odorless. He further argues, that, by reason of its antiseptic properties, salol calms the irritation of the stomach and bowels, and thereby reduces the temperature. The cream of tartar acts upon the kidneys and keeps the bowels open.

I watched the results of this treatment with keen interest, sincerely trusting that the doctor's sanguine expectations would be realized, yet I had serious misgivings on account of the nature and composition of the drug (salol).

The fact seems to have been overlooked, that salol is an irritant to the mucous membranes, it is not soluble in water, and but very slightly soluble in the stomach, but is decomposed by the pancreatic juice into salicylic acid and carbolic acid, the drug being a composition of salicylic acid 60, carbolic acid 40. The antiseptic virtues of the compound being, in all probability, due more to the latter, than to the former ingredient. The drug, when used in moderate doses, evidently has a beneficial effect in soothing the pain in the bowels, which is present in the great majority of cases. The beneficial results obtained by these moderate doses are, on the other hand, more than counterbalanced by the poisonous action of the drug when given so persistently and in such doses as were administered to the patients during this epidemic, regardless of the fact that poisoning by carbolic acid would certainly occur, owing to the decomposition of the drug by the pancreatic juice into its two original ingredients.

According to the best of the European, as well as American investigators, the drug is contraindicated in renal inflammation; especially in the acute form. Lepine and Dujardin Beaumetz condemn the administration of the salicylates in any form when albumen is present. Hesselbach maintains that salol is prone to affect the secreting structure of the kidney, and deeming it exceedingly dangerous when the kidney is diseased. He reports in the *Lancet* two fatal cases from the use of salol in such cases.

If salol be given in doses sufficient to reduce the temperature its poisonous effects are plainly made manifest, such as intense headache, restlessness, a profuse and exhausting sweat, delirium; in some cases the mental disturbance lasts for several days, the albumen in the urine increases, and the urine assumes a dark olive green color. These symptoms were well marked in several cases at this hospital, and two patients had strong and frequent epileptiform convulsions. Again, salol has a depressing influence over the circulation, and in yellow fever, capillary stagnation is a well marked feature, and the direct depression of the heart itself decreases the force of the circulation. In this latter view of the action of salol, I am sustained by Kolbe, Danewsky, Justi, Jaccoud, Ewald, Dujardin Beaumetz and Hesselbach.

After salol has been administered for any length of time, the urine becomes of a dark reddish brown color, and when nitric acid is added to it the albumen changes to a yellowish green color; this I have seen mistaken for bile, when, in fact, it was due to the union of carbolic acid and nitric acid, forming picric acid. ("Bile absent in yellow fever.") Salol, given in decided doses, whether in yellow fever or any other disease where the kidneys are the seat of an inflammatory process, is prone to bring about renal hemorrhages, and sometimes hemorrhagic cystitis.

Considering the decomposition of the drug into its two original ingredients, salicylic and carbolic acid, and the poisonous properties of the two acids, either alone or together, their pernicious action in irritating an already inflamed kidney, bringing about total suppression of urine, the most fatal of all symptoms in yellow fever, would certainly lead me to believe the drug to be contraindicated in this disease. Yet Dr. Nunez states that he has treated many cases successfully by this method, and he also assures me that A. A. Surgeon Hadra has treated the case at the General Hospital in the same way with very flattering results.

I do not deny that many cases have recovered under the salol treatment, they were, however, of a mild type, and would, in all probability, have passed through the ordeal with little medication, the disease being a self-limited fever. The records of this hospital show that the treatment of seven cases with salol was anything but satisfactory. In this statement I am fully sustained by the commanding officer of the hospital, and by the nurses who administered the medicine.

BAT SMITH, M. D.

